# Graph-based vision transformer with sparsity for training on small datasets from scratch

**DOI:** 10.1038/s41598-025-10408-0

**Published:** 2025-07-08

**Authors:** Peng Li, Lu Huang, Jin Li, Haiyan Yan, Dongjing Shan

**Affiliations:** 1 Emergency Department, Yantaishan Hospital, Yantai, Shandong 264008 China; 2 Southwest Medical University, Luzhou, 646000 China; 3 Shinawatra University, Bangkok, 10400 Thailand; 4 Yantai Municipal Health Service Center, Yantai, Shandong 264001 China; 5 School of Medical Information and Engineering, Southwest Medical University, Luzhou, 646000 China

**Keywords:** Vision Transformer, Graph convolution, Self-attention, Graph-pooling, Image classification, Biomedical engineering, Computer science

## Abstract

Vision Transformers (ViTs) have achieved impressive results in large-scale image classification. However, when training from scratch on small datasets, there is still a significant performance gap between ViTs and Convolutional Neural Networks (CNNs), which is attributed to the lack of inductive bias. To address this issue, we propose a Graph-based Vision Transformer (GvT) that utilizes graph convolutional projection and graph-pooling. In each block, queries and keys are calculated through graph convolutional projection based on the spatial adjacency matrix, while dot-product attention is used in another graph convolution to generate values. When using more attention heads, the queries and keys become lower-dimensional, making their dot product an uninformative matching function. To overcome this low-rank bottleneck in attention heads, we employ talking-heads technology based on bilinear pooled features and sparse selection of attention tensors. This allows interaction among filtered attention scores and enables each attention mechanism to depend on all queries and keys. Additionally, we apply graph-pooling between two intermediate blocks to reduce the number of tokens and aggregate semantic information more effectively. Our experimental results show that GvT produces comparable or superior outcomes to deep convolutional networks and surpasses vision transformers without pre-training on large datasets.

## Introduction

Transformer is a type of deep-neural network mainly based on self-attention mechanism which has achieved remarkable successes in sequence modelling tasks, such as natural language processing (NLP)^1^, video analysis^2^ and document generation^3^. Vision transformers (ViTs)^4^ are built upon the encoder architecture of Transformer and have become versatile methods applied to a multitude of tasks in computer vision, such as image classification^5,6^. ViTs tend to produce better image recognition performance than convolutional neural networks on large training datasets, with multi-layer perceptron (MLP) adopted for feature representation, which only has itself as a receptive field. Therefore, they rely on the multi-head self-attention (MHSA) module to capture the relationship between tokens. When trained with less data, lower attention layers are unable to focus on neighboring tokens and aggregate local information in the early stage^7^, which greatly affects the representation pipeline. As capturing local features in lower layers is crucial for the whole process, ViTs exhibit a significant performance gap compared to CNNs when trained from scratch on small datasets. For instance, the vanilla ViT model must first be pre-trained on the huge dataset JFT-300M^4^, and then fine-tuned on the common dataset ImageNet-1K^8^. When trained from scratch on ImageNet-1K alone, the accuracy is much lower^9^. However, in practice, most datasets are smaller than ImageNet-1K, and not all researchers can handle the task of pre-training a model on such a large dataset.

Additionally, it is important to strike a balance between the number of parameters and the representation power of the model for small dataset training. Deep learning tends to adopt small amount of parameters in order to be trained sufficiently on small datasets independent of large-scale pre-training on other datasets, indicating a small embedding size and hidden size are used for the models such as Transformer. However, a study in^10^ has shown that reducing the head size to a value below the input sequence length harms the representation power of each head, as a smaller head size imposes a rank constraint on the projection matrices in each head. Talking-heads or expanding hidden size^11^ are effective ways of raising the rank, but they can not be used directly in the models tackling small datasets and interaction among attention-heads should be considered more deeply.

To address the above-mentioned issues, we propose a graph-based vision transformer (GvT) that progressively abstracts features through three synergistic stages: (1) Graph convolution for Q/K injects local inductive bias via spatial-semantic adjacency; (2) Self-attention for S intentionally breaks sparse constraints to capture global dependencies; (3) Talking-heads-optimized sparse relation matrices guide graph convolution for V, integrating local adjacency and global semantics into semantic subgraphs. Our contributions are as follows: 1) An image-as-graph mechanism leverages graph convolutional projection to learn node dependencies, enhancing local feature attention in early layers; 2) To resolve low-rank bottlenecks, we incorporate talking-heads with sparse attention tensor selection, eliminating redundancy while enhancing expressive power; 3) Computational complexity is analyzed and validated via ablation studies. Extensive experiments demonstrate state-of-the-art performance across datasets without large-scale pre-training.

## Related work

Transformers^12^ have been proven to be more successful than convolutional and recurrent networks in a variety of sequential tasks with large-scale pre-training. Vision transformers (ViTs), variations of the transformer, have also achieved remarkable results on vision tasks. In order to improve the modeling of the relationships between image patches, new variations of Transformer have been proposed. For examples, some investigated token embedding: CPVT^13^ used conditional position encodings instead of positional embedding, Swin Transformer^14^ built hierarchical feature maps by merging image patches in deeper layers and performed computation of self-attention only within each local window. KGANet^15^ comprises a cross-layer information fusion module and a knowledge-guided attention module within a transformer backbone, for predicting the perceptual quality of an image without any reference information.

Others have explored convolution or graph convolution in their frameworks: CvT^16^ introduces a convolutional token embedding that performs overlapping convolution on token maps without positional encodings; GraphTrans^17^ employs a neural architecture with a Transformer subnetwork stacked on top of a GNN (e.g., GCN) layer stack. More recently, HiGDA^18^ proposes a hierarchical graph framework for semi-supervised domain adaptation, constructing local graphs to model patch-level dependencies within images and global graphs to aggregate category-level features across domains. Vision GNN^19^ and Vision HGNN^20^ treat images as graph-structured patches: the former utilizes multiple blocks of graph convolution modules, while the latter advances to hypergraph modeling via dynamic fuzzy clustering (e.g., Fuzzy C-Means) to capture higher-order patch relationships. These models rely on modular stacking or parallel integration of graph and Transformer components, fundamentally distinct from our approach that embeds graph convolutional projection directly within Transformer blocks. This design enables end-to-end feature learning where graph operations (e.g., adjacency matrix construction via spatial-semantic similarity) are deeply integrated into the Transformer’s attention mechanism, rather than existing as separate processing stages. Such integration mitigates the redundancy of traditional graph-based models (e.g., quadratic edge complexity in Vision GNN) and enhances inductive bias for small-data scenarios, as validated by our computational complexity analysis.

Despite the fact that ViTs trained on larger datasets perform well, they cannot compete with standard convolutional neural networks (CNNs) when trained on much smaller datasets like PET or CIFAR-100. To address this problem, work^21^ proposed a self-supervised training strategy and a loss function to improve performance. CCT^22^ utilized a convolutional tokenization module and replaced the class token with a final pooling sequence. DHVT^9^ introduced a new local constraint called patch alignment, which encouraged the local patches within the same block to align with each other, and enhanced representation interactions by introducing a self-attention mechanism, allowing the model to attend to important regions within the image. Although the previous works represent significant step forward in bridging the gap between CNNs and ViTs on small datasets, they fail in performance when compared with strong CNNs, and lack analysis of rank constraint on the projection matrices in each head brought by small hidden size.

Yang et al.^23^ have studied the effect of rank constraint caused by the small projection sizes in computing the softmax loss. The situation in self attention layers is a bit different. Bhojanapalli et al.^10^ pointed out that while the expressive power of each head reduced with the decreasing head size, the number of heads increases, which can potentially enhance the overall capacity of the layer. They also proved that reducing the head size to a value below the input sequence length harmed the representation power of each head, and then proposed to set them equal. Vaswani et al.^24^ noted that taking more attention heads projected to lower dimensionality made the dot product of query-vectors and key-vectors can no longer constitute an informative matching function. For addressing this problem, Shazeer et al.^11^ proposed a talking-heads attention that inserts a learned linear projection across the attention-heads dimension of the attention tensor.

Additionally, Pan et al.^25^ present a novel Cascade Hierarchical Graph Convolutional Network (Cas-HGCN) approach for detecting image manipulation. This approach incorporates a Feature Correlations Modeling (FCM) module, which effectively captures the intricate feature correlations between manipulated and non-manipulated regions across various scales, without the need for pre-training on large datasets. The key distinctions of our approach lie in its utilization of self-attention mechanisms within graph convolution to capture the global dependencies across the segmented patches. In contrast, the Cas-HGCN relies solely on cascading graph convolutions and falls short in addressing the over-smoothing issue that often accompanies graph convolutions.

## Neural architecture

**Fig. 1:**
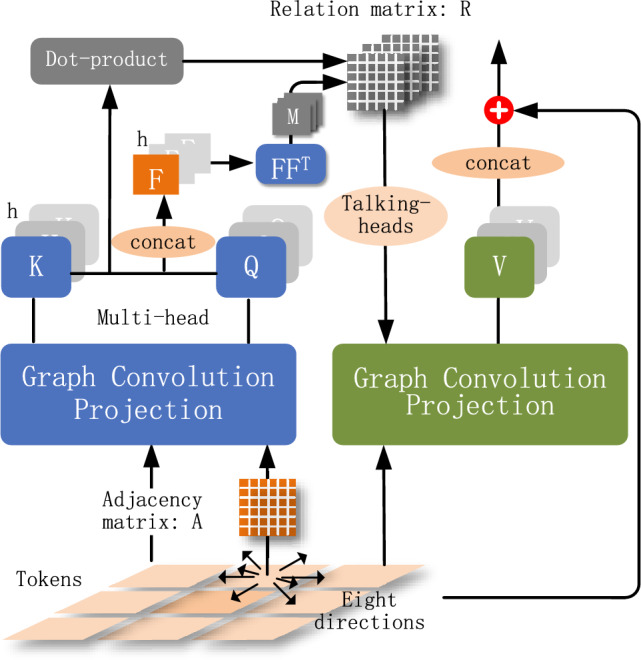
Pipeline of each residual block in the Graph-based vision Transformer (GvT). The block comprises: 1) Convolutional Token Embedding for local feature extraction; 2) Graph Convolutional Projection for computing queries and keys via spatial-semantic adjacency; 3) Scaled Dot-Product Attention generating initial attention scores; 4) Talking-Heads Component acting as a bridge between attention computation and graph-based feature aggregation. This module mitigates low-rank bottlenecks via sparse selection of attention tensors and enables cross-head information flow; 5) Graph Convolution Guided by Relation Matrix to compute values, incorporating global dependencies; 6) Residual Connection preserving high-frequency features.

GvT employs multiple repeated blocks, and the pipeline of a single block is depicted in Fig.1. The architecture of each block is detailed as follows.

### Convolutional token embedding

The convolutional token embedding in GvT seeks to model local contexts of tokens, ranging from low-level edges to higher order semantic primitives, over a two-layer convolutional network. Specifically, given a 2D image, a function $f(\cdot )$ is learned that maps each segmented image patch into a token; $f(\cdot )$ is a 2D group convolution operation^26^ with a kernel size of 5, which shares convolutional kernel among different groups of feature channels and can reduce memory requirements. The convolutional token embedding layer introduces a local inductive bias for each patch, permitting the adjustment of the token feature dimension. Generally, the model will be more effective when capturing the semantic context of the tokens more accurately.

### Calculation of queries and keys

In the pipeline shown in Fig.1, the token map input into each residual block should be interpreted as graph data and then fed into the graph convolution layer. In other words, an adjacency matrix needs to be learned to indicate token-wise relations in the maps. An initial adjacency matrix $A_I$ is created by recording the eight-directional neighboring relations of the tokens in the map, and then the context similarity in the feature space must be taken into account since the matrix $A_I$ only considers a coarse form of spatial relations.

The tokens are then defined as graph nodes: $V={v_1,v_2,\ldots ,v_n }\in \mathbb {R}^{n\times d}$, each with *d* dimensional features. The graph is then represented as $G={V\in \mathbb {R}^{n\times d}, E\in \mathbb {R}^{n\times n}}$, where *E* denotes the edges of the graph and is defined as:

1 $$\begin{aligned} \begin{aligned}&E_{v_{ij}}= \frac{\exp {(D_{cos}(v_i,v_j))}}{\sum _{k=1}^{n}{\exp {(D_{cos}(v_i,v_k))}}}, \end{aligned} \end{aligned}$$

where $E_{v_{ij}}$ is the similarity degree between the nodes $v_i$ and $v_j$, $i,j\in [1,2,\ldots ,n]$. $D_{cos}$ is cosine distance measuring the similarity score of a pair of nodes, which can be defined as:

2 $$\begin{aligned} \begin{aligned}&D_{cos}(v_i,v_j) = 1 - \frac{v_i\cdot v_j}{||v_i|| ||v_j||} , \end{aligned} \end{aligned}$$

the cosine distance is used to calculate the similarity scores between the node pairs, where $\cdot$ and $||\cdot ||$ denote element-wise multiplication and $\ell _2$ norm, respectively. This formula ($1-*$) guarantees that the more similar the node pair is, the smaller the score is. Therefore, the sum of the initial adjacency matrix $A_I$ with an identity matrix *I* is modified by considering the similarity scores of the tokens (*E*). The final adjacency matrix is formulated as:

3 $$\begin{aligned} \begin{aligned}&A = (A_I+I)\odot E, \end{aligned} \end{aligned}$$

where $\odot$ denotes element-wise multiplication. $(A_I+I)\odot E$ represents that the initial adjacency matrix $A_I$ is summed with an identity matrix *I*, and then modified by considering the similarity scores of the tokens (*E*).

Next, queries and keys can be calculated using graph convolution projection:

4 $$\begin{aligned} \begin{aligned}&x^{q/k}= G^{q/k}_{A}(x) = (D_A^{-\frac{1}{2}}AD_A^{-\frac{1}{2}})xW_{q/k}, \end{aligned} \end{aligned}$$

where *x* is the input token map of one transformer block, $D_A\in \mathbb {R}^{n\times n}$ are the degree matrix of the adjacency matrix *A*, $W_{q/k}\in \mathbb {R}^{d\times d}$ are learnable parameters used for linear projection. In practice, $x^{q/k}$ are split into *h* heads averagely, with each head $(x_i^{q/k}, i=1,\ldots ,h)$ responsible for learning a different representation, and accordingly the weights $W_{q/k}$ are replaced with *h* number of small weights $W_{q/k,i}\in \mathbb {R}^{(d/h)\times (d/h)}$. $G_A(\cdot )$ denotes graph convolution operation performed on the adjacency matrix *A*.

### Calculation of values

The scaled dot-product on queries and keys is performed to create attention tensor *S* and can be formulated as follows:

5 $$\begin{aligned} \begin{aligned} S_i&= Attention(x_i^{q},x_i^{k}) = softmax\left( \frac{x_i^{q}(x_i^{k})^{\textrm{T}}}{\sqrt{d}}\right) , \end{aligned} \end{aligned}$$

where *d* denotes feature dimension of the queries and keys, $S_i\in \mathbb {R}^{n\times n}$ represents the attention score calculated on *i*-th heads and stores the attention scores assigned to each token. The increase in the number of heads seemingly enhance the model’s expressive power, but it also reduces the head size and thus diminishes its expressive power. If the number of heads *h* exceeds $\frac{d}{n}$, the attention unit within each head will project onto a dimension smaller than *n*, resulting in a low-rank bottleneck that impairs its ability to represent arbitrary context vectors^10^. In other words, A rank-deficient $S_i$ cannot uniquely assign attention scores to all *n* tokens. Redundant columns (token relationships) arise, reducing discriminative power. For example, if column *j* is a linear combination of columns *k* and *l*, the model cannot attend distinctly to token *j*.

#### Talking-heads integration for low-rank bottleneck mitigation

For addressing the low-rank bottleneck, Shazeer et al.^11^ propose a talking-heads attention that includes linear projections across the attention-heads dimension. Nevertheless, according to Eq. (5), it can be deduced that the attention tensor should be full column rank if the queries of the tokens are different from each other. Due to the low-rank constraint, a majority of the columns of the attention tensor are repeating the precise attention scores using linear correlation and can not represent the attention scores of a specific token correctly. Thus, those columns should be removed from the attention tensors, with the remained tensors used for head talking in the next step. This is achieved by conducting sparse selection on the attention tensor, resulting a relation matrix. First, we have $f_i=[x_i^q,x_i^k]W_h$, where $W_h\in \mathbb {R}^{(2d/h)\times h}$ symbolizes a fully connected layer, then we have $F=[f_1,f_2,\ldots ,f_h]\in \mathbb {R}^{h\times n}$, which denotes feature extracted from concatenated queries and keys. The bilinear pooled feature $C=FF^{\textrm{T}}\in \mathbb {R}^{h\times h}$, with each row representing the correlation of one feature channel (from one head) with other channels, captures the second-order statistics and can be used to select out the maximal linearly independent array of the attention matrix from each head. By using a linear layer, $Y=CW_c\in \mathbb {R}^{h\times n}$ evolves to a matrix with *n* columns, where $W_c\in \mathbb {R}^{h\times n}$.

To generate a sparse matrix, we first apply a shrinkage function to matrix $Y$, yielding $Z = f_{\text {shr}}(Y)$. Here, $f_{\text {shr}}(x)$ is defined as the sigmoid function applied to $x \odot \frac{1}{u}$ (see Fig. 3), where $u$ is a trainable vector initialized with elements as 1 to adjust the function slope, and $\odot$ denotes element-wise multiplication. Next, we reshape $Z \in \mathbb {R}^{h \times n}$ into a diagonal matrix $M \in \mathbb {R}^{h \times n \times n}$ by transforming the last column of $Z$ into a square diagonal array. This transformation yields the diagonal matrix *M*, whose construction process is formalized as follows.

Sparse Diagonal Matrix $M$ Construction Steps:

1) Compute $Y = CW_c$ via linear projection;

2) Apply shrinkage: $Z = \text {sigmoid}(Y \odot u^{-1})$, where $u$ is a trainable slope-adjustment vector (initialized as 1);

3) Reshape $Z$ to form a diagonal matrix $M$ with dimensions $\mathbb {R}^{h \times n \times n}$.

After that, *M* can be used to select out valuable rows and columns (they are different as to different heads) in the attention tensors and keep the tensors symmetric:

6 $$\begin{aligned} \begin{aligned} {\hat{S}} = M\times S\times M, \end{aligned} \end{aligned}$$

where the symbol $\times$ denotes matrix multiplication in batch mode, $S=[S_1,S_2,\ldots ,S_h]\in \mathbb {R}^{h\times n\times n}$.

Finally, we can generate relation matrix for head talking:

7 $$\begin{aligned} \begin{aligned} \begin{pmatrix} R_1\\ R_2\\ \vdots \\ R_h \end{pmatrix}=\Phi \begin{pmatrix} \hat{S_1}\\ \hat{S_2}\\ \vdots \\ \hat{S_h} \end{pmatrix} \end{aligned} \end{aligned}$$

where $\Phi \in \mathbb {R}^{h\times h}$ is a learnable parameter matrix, and $R_i$ represents the interactive relation matrix using linear projections across the attention heads. As we know, $x_i^q$ and $x_i^k$ in Eq. (5) have 2*nd*/*h* parameters in total, and encounter low rank bottleneck when generating attention tensor $S_i$ with $n^2$ parameters. Head talking can increase the rank by sharing maximal linearly independent subset across the heads.

**Fig. 2:**
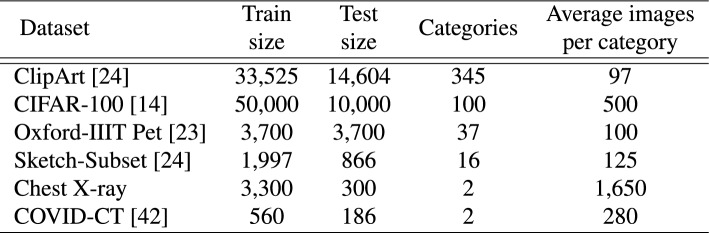
The statistics of training datasets. The train and test size of each dataset, including the number of categories and average images per category in training sets are reported.

**Fig. 3:**
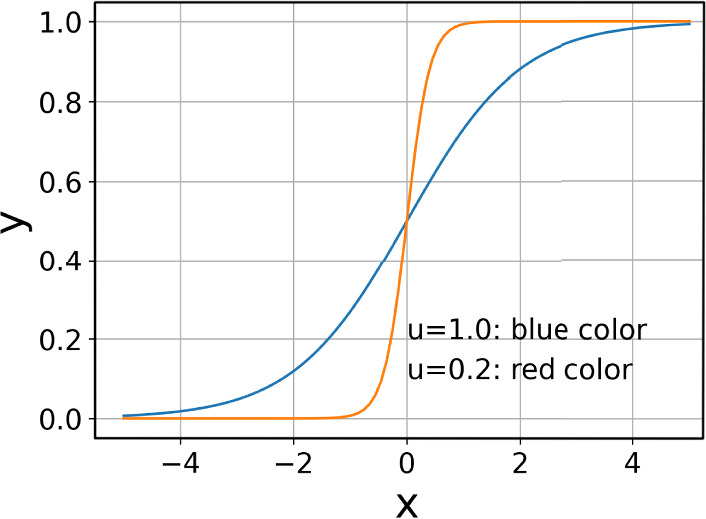
Curves of $$y=$$ $$f_{shr}(x|u=1.0\;or\;0.2)$$.

#### Graph convolution guided by talking-heads relation matrix

Instead of using the weighted sum of the projected tokens, we can use graph convolution projection to calculate values based on the tokens and the relation matrix. This form of global attention incorporates stronger inductive biases, helping to capture more non-local dependencies and thereby providing richer contextual information of the neighborhood, resulting in learning more reliable representations. Calculation of the values is shown as follows:

8 $$\begin{aligned} \begin{aligned}&x_i^{v}= G^{v}_{{R}_i}(x_i) = (D^{-\frac{1}{2}}{R}_iD^{-\frac{1}{2}})x_i W_{vi} + x_i, \end{aligned} \end{aligned}$$

where $D\in \mathbb {R}^{n\times n}$ are the degree matrix of the relation matrix ${R_i}$, $x_i$ and $W_{vi}\in \mathbb {R}^{(d/h)\times (d/h)}$ are input features and learnable parameters related to one attention head, respectively. The subscript *i* represents head index, $G_{{R}_i}(\cdot )$ denotes graph convolution operation performed on the relation matrix ${R}_i$. The output features computed in different heads are concatenated to one vector for final classification using a linear layer. By incorporating the graph convolution layer, the model is able to incorporate the neighbor relations and context into the learning process and to update the token representations accordingly. This helps the model to better capture global (long-term) dependencies and to learn more meaningful representations.

### Graph-pooling

As the input images are divided into tokens based on grid structure, rather than their semantic content, there is a risk of fragmenting semantic regions in an unreasonable manner. To address this issue, we propose the use of graph-pooling operations^27^ to reduce the number of tokens and aggregate semantic information more effectively. Given the input matrix $X=[x_1,x_2,\ldots ,x_n]^{\mathrm{{T}}}\in \mathbb {R}^{n\times d}$, representing inputs to each transformer block, the second-order pooling can be formulated as: $X'=UX^{\mathrm{{T}}}X\in \mathbb {R}^{t\times d}$. Here, $U\in \mathbb {R}^{t\times t}$ is a transformation matrix. For the adjacency matrix *A*, we have $C=UX^{\mathrm{{T}}}\in \mathbb {R}^{t\times n}$, and the new adjacency matrix $A'=CAC^{\mathrm{{T}}}\in \mathbb {R}^{t\times t}$.

The computational complexity of GvT can be significantly reduced compared to ViT. In ViT, each block consists of one MSA module and one feedforward module, with computational complexities of $4nd^2+2n^2d$ and $6nd^2$, respectively, where *n* and *d* represent the number of input tokens and hidden units. In GvT, the three graph convolution operations cost $3(n^2d+nd^2)$, the calculation of attention tensor *S* costs $n^2d$, and the modification of *S* (related to Eqn(6)) cost $2n^3$ (We omit computation of the matrixes *W* and *M* in determining complexity as they are linear to *n* or *d*). For easy comparison, we assume that *n* is approximately equal to *d* (for example, both *n* and *d* are set to 64 in some parts of our experiments), with *u* representing their mean value. Therefore, we have:

9 $$\begin{aligned} \begin{aligned} \Omega (ViT)=10nd^2+2n^2d\approx 12u^3,\\ \Omega (GvT)=3nd^2+4n^2d+2n^3\approx 9u^3, \end{aligned} \end{aligned}$$

which implies that when the token number *n* is smaller than the hidden size *d*, the computational complexity is reduced compared to ViT. In our experiments, we applied graph pooling between two intermediate blocks and reduced *n* to *t*.

## Experiments

In this section, we evaluate the GvT model on two tasks: image classification and graph structural data classification. At first, the image classification tasks are conducted by training from scratch on small datasets, such as ClipArt^28^, CIFAR-100^29^, Oxford-IIIT Pet^30^, Sketch^28^, Chest X-ray and COVID-CT^31^. These datasets have a limited amount of training data overall and a limited amount of training data for each category, the details are shown in Table 2. We compare the model to different deep convolutional networks, including ResNet^32^, VGGNet^33^, TCN^34^, and also the transformer variants: ViT^35^, Swin Transformer^14^, Talking Heads^11^, GraphTrans^17^, Vision GNN^19^, Vision HGNN^20^ and DHVT^9^). All the models are trained from scratch on small datasets.

Additionally, we evaluate the GvT model on graph-structured data for semi-supervised document classification in citation networks, closely following the experimental setup described in Yang et al.^36^. Dataset statistics are summarized in Table 1. For benchmark datasets Citeseer, Cora, and Pubmed^37^, nodes represent documents and edges denote citation links. The label rate is defined as the ratio of labeled nodes used for training to the total number of nodes in each dataset, capturing the scarcity of annotated data typical in such semi-supervised scenarios.

**Table 1:** Comparison of citation network datasets.

Dataset	Nodes	Edges	Classes	Features	Label
					rate
Citeseer	3,327	4,732	6	3,703	0.036
Cora	2,708	5,429	7	1,433	0.052
Pubmed	19,717	44,338	3	500	0.003

For all comparative models in the first task, we either re-ran the code provided by the authors or directly used their implementations, except for Talking Heads and GraphTrans. These two methods were implemented based on the ViT framework, where GraphTrans incorporates a 3-layer GCN with residual connections. Unless specified otherwise, images are first divided into 64 tokens before entering the bottom block, and then pooled to 16 tokens prior to the middle block (the fifth block in a 7-block architecture). Convolutional networks were trained for 100 epochs, while transformer variants required 300 epochs due to their slower convergence. For the second task, the results of all comparative baselines were derived from the Planetoid paper^36^ or GCN^38^. Our proposed method employed 2 blocks without a graph pooling operation, due to the purpose of node-level classification.

In GvT, we use AdamW^39^ optimizer with a weight decay of 0.05, an initial learning rate of 5*e*−4 with a cosine learning rate decay scheduler, and a batch size of 64. The training is performed on they converge relatively slowly. Both trainings are performed on two GPUs parallelly: one NVIDIA GeForce RTX 2080Ti and one NVIDIA T600 Laptop GPU. Models are evaluated using five independent runs, with the highest accuracies recorded and averaged over these five runs for comparison purposes. The time spent on training is measured in terms of floating point operations (FLOPs), while the speed of inference is indicated by Frames Per Second (FPS).

**Table 2:** Comparative results of classification on three small vision datasets, with accuracy rate (%) adopted as evaluation metric.

$\hbox {Model}^a$	ClipArt				CIFAR-100				Oxford-IIIT Pet			
	Params	FLOPs	FPS	Acc	Params	FLOPs	FPS	Acc	Params	FLOPs	FPS	Acc
ResNet	209K	266.8M	556	28.25	507K	866.6M	1127	48.98	209K	266.8M	556	20.41
VGGNet	253K	182.1M	554	34.30	660K	1.14G	1037	55.39	253K	182.1M	554	24.15
TCN	172K	270.9M	114	13.26	494K	1.19G	886	35.46	172K	270.9M	114	10.50
ViT	271K	30.5M	207	22.64	811K	51.6M	1064	50.61	271K	30.5M	207	14.87
Swin T	269K	49.2M	551	33.92	550K	113.7M	731	55.49	269K	49.2M	551	28.36
Talking Heads	212K	26.7M	415	24.16	651K	72.7M	740	51.84	212K	26.7M	415	14.11
GraphTrans	251K	14.6M	561	26.15	822K	51.5M	1273	56.42	251K	14.6M	561	14.74
Vision GNN	207K	30.8M	312	23.49	524K	101.92M	437	54.13	207K	30.8M	312	22.49
Vision HGNN	274K	– –	17	27.46	518K	– –	5	57.13	274K	– –	17	25.39
DHVT	442K	30.1M	214	31.58	555K	37.4M	536	52.74	442K	30.1M	214	14.47
Ours (GvT)	173K	21.4M	560	$\mathbf {35.12}$	503K	51.2M	1136	$\mathbf {58.16}$	173K	21.4M	560	$\mathbf {30.66}$

^a^ The parameter numbers take approximate values and are kept roughly the same or less than ours for fair comparison.

### Performance comparisons

#### ClipArt

The ClipArt^28^ dataset contains 33,525 training images and 14,604 testing images, divided into 345 different object categories, with an average of 97 images per category. Table 2 displays the model size and accuracy, with all models having roughly the same number of parameters. TCN^34^ is a comparative method that uses temporal convolutional methods to reshape 2D images into sequential data for classification. Vision GNN^19^, which models images as sparse graphs with pairwise edges, achieves 23.49% accuracy with 207 K parameters and 30.8M FLOPs. Its performance is constrained by redundant edge connections, leading to suboptimal feature aggregation for complex object patterns in ClipArt. In contrast, Vision HGNN^20^ leverages hypergraphs to capture higher-order patch relationships via Fuzzy C-Means clustering, improving accuracy to 27.46%. However, this approach incurs significant computational overhead, evident in its low inference speed (17 FPS) due to dynamic hyperedge updates.

Our method GvT uses 7 residual blocks with hidden size set as 64 and head number set as 8. GvT outperforms TCN on ClipArt and significantly improves over ViT^35^ and DHVT^9^, surpassing ResNet^32^ and behaving better than the performance of VGGNet^33^ and Swin Transformer^14^. GvT also exhibits similar inference speech as convolutional networks, yet with significantly fewer FLOPs.

#### CIFAR-100

The CIFAR-100 dataset^29^ contains 60,000 32$\times$32 color images in 100 classes, each with 600 images divided into 500 for training and 100 for testing. In Table 2, ResNet and VGGNet achieve testing accuracy scores of 48.98% and 55.39%, respectively, on the CIFAR-100 classification task. Our method GvT uses 7 residual blocks with hidden size set as 128 and head number set as 8. The original vision of ViT does not have good results as it needs to be pre-trained on larger datasets. Talking Heads and DHVT works better than ViT while GvT improves significantly over ViT and achieves the highest testing accuracy by incorporating graph convolutional operations. Likewise, GvT boasts a fast inference speed comparable to that of ResNet and VGGNet. These performance scores show that GvT is capable of outperforming classic convolutional networks on the CIFAR-100 dataset, and surpasses other ViT variants, making it an attractive architecture for computer vision projects.

**Table 3:** Comparative results of classification on three small datasets, with accuracy rate (%) adopted as evaluation metric.

Model	Sketch-Subset				Chest X-ray				COVID-CT			
	Params	FLOPs	FPS	Acc	Params	FLOPs	FPS	Acc	Params	FLOPs	FPS	Acc
ResNet-50	102K	198M	269	$\mathbf {66.40}$	102K	198M	269	83.96	69K	190.8M	318	75.26
VGGNet-19	95K	205M	283	65.01	95K	205M	283	84.33	71K	189.4M	309	74.19
TCN	107K	191M	169	49.14	107K	191M	169	68.33	82K	146.2M	131	60.32
ViT	152K	9.8M	261	51.26	181K	11.7M	246	80.67	102K	6.5M	103	70.43
Swin T	107K	24.0M	128	62.12	129K	28.9M	106	83.67	63K	14.3M	184	78.49
Talking Heads	93K	16.0M	107	54.50	174K	27.5M	66	81.79	69K	14.4M	98	72.04
GraphTrans	151K	9.7M	263	54.04	151K	9.7M	263	69.33	102K	6.5M	103	69.35
Vision GNN	124K	30.6M	326	56.24	124K	30.6M	326	82.18	124K	30.6M	326	74.18
Vision HGNN	274K	– –	17	57.05	274K	– –	17	80.35	274K	– –	17	79.26
DHVT	189K	13.4M	227	52.17	189K	13.4M	227	73.33	67K	4.5M	274	65.59
Ours (GvT)	87K	13.5M	213	63.39	101K	16.4M	197	$\mathbf {87.00}$	54K	12.3M	226	$\mathbf {83.33}$

#### Oxford-IIIT pet

Oxford-IIIT Pet is a dataset of pets categorized into 37 categories with around 100 training images for each category. The total number of images and the number of images per category are both small. The images have a large variations in scale, pose and lighting, each associated with a label. Testing using this dataset is challenging for Transformers since there are fewer images per category. In Table 2, the models used for comparison have around 100 K parameters. Our method GvT uses 7 residual blocks with hidden size set as 64 and head number set as 8. GvT performs better than existing classic methods, including convolutional networks which underperform with small datasets like this.

The majority of the comparative models achieve accuracies below $20\%$ except for the VGGNet and Swin Transformer, while GvT achieves the highest. The results show that GvT is a promising approach for small datasets, which are often difficult to work with. The traditional approaches of using convolutions are not always effective for these types of datasets, as they require a large number of training images to learn features that can generalize well. GvT, on the other hand, is a method that can learn to extract useful features from a small number of images. It accomplishes this by integrating information from different sources, such as semantic information, visual features, and context. This approach allows GvT to learn more robust representations, even with limited data.

#### Sketch-subset

We select a subset from the Sketch^28^ dataset, which contains the former 16 categories, with 1,997 training images and 866 testing images. Information about model sizes and classification accuracies are presented in Table 3. Our method GvT uses 12 residual blocks with hidden size set as 40 and head number set as 5. When using 64 tokens, GvT approaches the performance of ResNet and VGGNet. When using 196 tokens, i.e., the images are resized to shape of $224\times 224$ and split into $14\times 14$ patches (tokens), GvT takes 9 residual blocks with hidden size set as 32 and head number set as 8 for keeping the similar parameters, achieving the best accuracy (67.55%). The FLOPs of GvT remains consistent with those of transformer variants, yet significantly lower than those of convolutional networks, suggesting a much faster training process.

#### Chest X-ray

Medical imaging plays a crucial role in accurately diagnosing and treating various health conditions. Chest X-rays^40^ are commonly used to detect and diagnose respiratory and cardiac problems, but interpreting them can be difficult even for experienced radiologists due to the complexity of the images and the risk of misinterpretation. To overcome this challenge, artificial intelligence (AI) and machine learning techniques are being applied to classify chest X-ray images and improve diagnostic accuracy. Machine learning algorithms, such as convolutional neural networks (CNNs), have been successfully used to classify chest X-ray images as either normal or abnormal, and can even identify specific subcategories like pneumonia, tuberculosis, or lung cancer. However, there are still challenges to be addressed, such as the lack of diverse samples in the chest X-ray dataset, which requires expert labeling.

The dataset for chest X-rays, which we adopted, contains 3,600 images of both normal and abnormal cases with 1,800 images in each category. Among these, 1,600 images are used to train the model while the rest are used for testing. All images were adjusted to a size of 256$\times$256 pixels. Information about model sizes and classification accuracies can be found in Table 3. GvT uses 10 residual blocks with hidden size of 48 and head number of 8, results indicate that our proposed GvT model has the highest accuracy at 87.00%, followed by VGGNet-19 at 84.33%. DHVT performed slightly better than TCN, which achieved the lowest accuracy at 68.33%. These findings suggest that deep learning models can be effective in diagnosing lung nodules from Chest X-Ray images, with GvT being the most accurate model currently.

**Table 4:** Summary of results in terms of classification accuracy (in percent %).

Method	Citeseer	Cora	Pubmed
DeepWalk^41^	43.2	67.2	65.3
ICA^42^	69.1	75.1	73.9
Planetoid^36^	64.7	75.7	77.2
GCN^38^	70.3	81.5	79.0
GvT (WoT Graph pooling)	72.1	82.3	80.5

#### COVID-CT

COVID-CT^31^ dataset is a valuable resource for developing and testing artificial intelligence models for COVID-19 diagnosis. The limited availability of COVID-19 cases for training and evaluation means that models trained from scratch on small datasets can potentially accelerate COVID-19 detection. The COVID-CT dataset comprises 349 CT images labeled as positive for COVID-19 and 397 non-COVID-19 CT images serving as negative examples. These images are from 216 patient cases, have varying sizes, and are all resized to $256\times 256$ pixels. They are divided into training and testing sets, with 280 images per category used for training and the remaining images used for testing. Table 3 displays the results of comparison, and our model GvT has achieved the highest accuracy, indicating the effectiveness of GvT for this particular classification task. GvT uses 7 residual blocks with hidden size of 40 and head number of 5, it performs well on small datasets with a total number of 54*K* parameters, surpassing other models with accuracies under 76%.

**Fig. 4:**
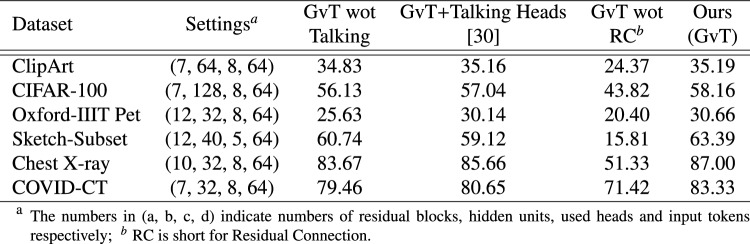
Comparative results (accuracy rate (%)) for ablation study on six small datasets.

**Fig. 5:**
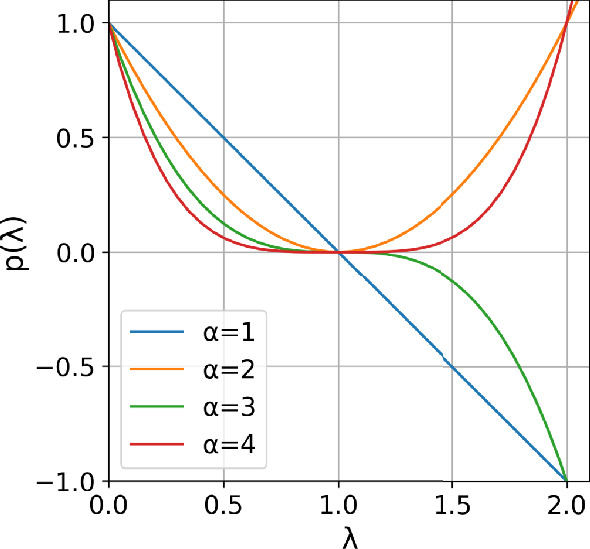
Curves of $$p(\lambda )$$.

**Table 5:** Comparison of GvTs on six small datasets, with different token numbers adopted and evaluated with accuracy rate (%).

Dataset	$\hbox {Settings}^a$	Params	Ours (GvT)	$\hbox {Settings}^a$	Params	Ours (GvT)
ClipArt	(7, 64, 8, $\textbf{64}$)	173K	35.12	(7, 56, 7, $\textbf{100}$)	163K	$\mathbf {35.24}$
CIFAR-100	(7, 128, 8, $\textbf{64}$)	503K	58.16	(12, 96, 8, $\textbf{100}$)	526K	$\mathbf {58.49}$
Oxford-IIIT Pet	(12, 56, 7, $\textbf{64}$)	192K	31.87	(9, 48, 6, $\textbf{196}$)	201K	$\mathbf {36.02}$
Sketch-Subset	(12, 40, 5, $\textbf{64}$)	87K	63.39	(9, 32, 8, $\textbf{196}$)	105K	$\mathbf {67.55}$
Chest X-ray	(10, 48, 8, $\textbf{64}$)	101K	$\mathbf {87.00}$	(12, 40, 5, $\textbf{100}$)	126K	86.67
COVID-CT	(9, 56, 7, $\textbf{64}$)	156K	82.82	(11, 48, 6, $\textbf{100}$)	155K	$\mathbf {85.48}$

^a^ The numbers in (a, b, c, d) indicate numbers of residual blocks, hidden units, used heads and the input tokens respectively.

#### Citation network

This task belongs to node-level classification, which involves predicting labels for each individual node rather than classifying the entire graph (e.g., the entire citation network) or edges (e.g., citation relationships). For example, in the Cora dataset (containing machine learning papers), each paper needs to be classified into one of seven categories such as “neural networks” or “probabilistic methods”. The node aggregation method of GvT based on attention scores complements GCN’s reliance on static connections by fusing semantic similarity and citation adjacency relationships, demonstrating practicality in semi-supervised scenarios with scarce labels that require leveraging semantic information. Classic models such as DeepWalk and graph embeddings were also used for performance comparison. In Table 4, experimental results validate the superiority of our approach in classification accuracy.

### Ablation studies

As previously explained, the GvT model consists of multiple self-attention residual modules that involve talking heads. The ablation study includes comparing the GvT models with and without incorporating talking heads, in which the method “GvT wot Talking Heads” indicates multiple attention modules without head interaction across heads, “GvT+Talking Heads^11^” means that GvT method adopts the Talking Heads mechanism proposed in the work^11^, i.e., a learned linear projection is used to combine the attention-heads. As comparison, GvT propose to remove linearly dependent rows and columns in the low rank relation matrix with sparse diagonal matrix.

The experimental results on the six small datasets are displayed in Table 4, aiming to prove the efficiency of our proposed talking-heads attention when encountering low-rank bottleneck. For comparison, the methods maintain the same architectural settings, which are presented in the second column of the table. Totally, “GvT+Talking Heads^11^” and GvT both improve over “GvT wot Talking”, and GvT behaves the best by using our proposed talking-heads approach. On ClipArt and CIFAR-100, GvTs using two kinds of talking heads preform similarly as the models’ hidden size is large than or equal to the token number, which avoids low-rank bottleneck, and talking heads can still help to improve performance for the information interaction among the heads.

Next, it is not hard to deduce that the relation matrix $R_i$ in Eqn(8) is symmetric, thus we have:

10 $$\begin{aligned} \begin{aligned} R_{sym}&=D^{-\frac{1}{2}}{R}_iD^{-\frac{1}{2}}\\ &=D^{-\frac{1}{2}}(D-L)D^{-\frac{1}{2}}=I-\tilde{L}, \end{aligned} \end{aligned}$$

where $\tilde{L}$ is a normalized Laplacian matrix and orthogonally diagonalizable: $\tilde{L}=U\tilde{\Lambda } U^\textrm{T}$, $\tilde{\Lambda }=diag[\lambda _1, \lambda _2,\ldots , \lambda _n]$. According to the minimax principle of rayleigh quotient^43^, the Eigen values of a positive semidefinite matrix $\lambda _i \in [0,2]$. Thus we have: $R_{sym} =I-\tilde{L} = U(I-{\tilde{\Lambda }})U^\textrm{T}$, then define a frequency response function as $p(\lambda )=1-\lambda \in [-1,1]$. Apparently, the function has linear shrinkage property which can amplify low-frequency components and compress high-frequency, i.e., acts as a low-pass filter to input graph signal (see Fig.5). When stacked multiple graph convolution layers, signal is multiplied by $R_{sym}$ repeatedly, frequency response function evolves to $p(\lambda )=(1-\lambda )^\alpha$, with $\alpha$ denoting the layer number. The function has stronger scaling ability in low frequency range, indicating a stronger low-pass property. Nevertheless, high-frequency components are also necessary for fine-grained classification of images, so that is why a residual connection is utilized in Eqn(8) to convey high-frequency directly. Experimental demonstration is presented in Table 4, in which GvT without residual connection ranks last in the classification tasks.

Finally, we present the comparison of GvTs with different token numbers in Table 5, which are usually set empirically. Models in the left part are trained by splitting the input images into 64 patches (tokens), and then those employing 100 or 196 tokens are used for comparison. Expanding the token numbers can make more refined segmentation on the images, the layer numbers combined with hidden size are adjusted to maintain similar number of parameters. The results demonstrate that this way shows potential in further promoting the model performance. Meanwhile, the demand for memory and computation power expands as the token number increases.

## Conclusions

This paper introduces a new architecture for vision transformers that uses graph convolutional projection and talking-heads attention (GvT). The GvT-based architecture can train from scratch on small datasets and reach state-of-the-art performance on a range of different datasets. The graph convolution operations allow the model to learn better feature representation by leveraging an inductive bias for attending to local features in the early stages and capturing relationships between tokens. Talking-heads share linearly independent group across multi-head attention and tackle constraint brought by low-rank bottleneck. Graph-pooling can aggregate semantic region and reduce the number of tokens. In experiments, we test the models on various image classification tasks, including regular images and specialized medical images, and conduct ablation study for analyzing efficiency of the adopted components. The results can demonstrate the performance and potential of GvT for training from scratch on these smaller datasets, which are often encountered in real-world applications, and future research will build upon these findings to further improve performance.

## Data Availability

The code for our proposed model is publicly available on the website: https://github.com/cvcoding/GvT.
